# Erosive Esophagitis in the Obese: The Effect of Ethnicity and Gender on Its Association

**DOI:** 10.1155/2016/7897390

**Published:** 2016-03-28

**Authors:** Albin Abraham, Seth Lipka, Rabab Hajar, Bhuma Krishnamachari, Ravi Virdi, Bobby Jacob, Prakash Viswanathan, Paul Mustacchia

**Affiliations:** ^1^Department of Gastroenterology, Nassau University Medical Center, East Meadow, NY 11554, USA; ^2^Department of Internal Medicine, Nassau University Medical Center, East Meadow, NY 11554, USA; ^3^Research Department, NYIT College of Osteopathic Medicine, Glen Head, NY 11545, USA

## Abstract

*Background.* Data examining the association between obesity and erosive esophagitis (ErE) have been inconsistent, with very little known about interracial variation.* Goals.* To examine the association between obesity and ErE among patients of different ethnic/racial backgrounds.* Methods.* The study sample included 2251 patients who underwent esophagogastroduodenoscopy (EGD). The effects of body mass index (BMI) on ErE were assessed by gender and in different ethnic groups. Odds ratios (ORs) and 95% confidence intervals (CIs) were calculated using multivariate logistic regression analysis.* Results.* The prevalence of ErE was 29.4% (661/2251). Overweight and obese subjects were significantly more likely to have ErE than individuals with a normal BMI, with the highest risk seen in the morbidly obese (OR 6.26; 95% CI 3.82–10.28; *p* < 0.0001). Normal weight Black patients were less likely to have ErE as compared to Caucasians (OR 0.46; 95% CI 0.27–0.79; *p* = 0.005), while the odds ratio comparing normal weight Hispanics to normal weight Whites was not statistically significant. No effect modification was seen between BMI and race/ethnicity or BMI and gender. Significant trends were seen in each gender and ethnicity.* Conclusions.* The effect of BMI on ErE does not appear to vary by race/ethnicity or gender.

## 1. Introduction

The prevalence of obesity, which has been defined as a body mass index (BMI) greater than 30 kg/m^2^, has grown to epidemic proportions in the Western world, with an estimated $147 billion spent in annual healthcare costs [1]. Associated complications from obesity have increased morbidity and mortality and have significantly decreased quality of life in such individuals [2, 3]. Data from the latest National Health and Nutrition Examination Survey (NHANES) indicate that the prevalence of obesity increased from 15% in 1976–80 to 35.7% in 2009-10 [4, 5]. The highest rates of age adjusted obesity were found in non-Hispanic Blacks (49.5%) compared to non-Hispanic Whites (34.3%) and Hispanics (39.1%) [4]. Similarly, the prevalence of gastroesophageal reflux disease (GERD) has been gradually rising in the United States, affecting up to 45% of the population, with at least 20% reporting weekly symptoms [6–9]. As a result, GERD imposes a tremendous burden on health care and in 2004 accounted for $12 billion in healthcare expenditure [6].

Overall, though few studies have yielded conflicting results, obesity has been shown to be positively associated with GERD [5, 10–19]. However, the association between obesity and erosive esophagitis (ErE) has only been considered recently, and studies have yielded inconsistent results [11, 17, 20–26]. Hampel et al. [27] showed that obesity is overall associated with a significant risk of ErE, though a more recent meta-analysis demonstrated a strong association in males, but not in females [28]. Though ErE has been reported to be more common among Caucasians [9, 29, 30], very little is known about effects of race on the association between increasing BMI and ErE, with no published data on the emerging Hispanic population. The objective of our study was to investigate the impact of obesity on ErE in different genders and in various ethnic backgrounds, especially among the rapidly increasing Hispanic and Black population.

## 2. Methods

### 2.1. Study Design and Population

A retrospective case control study was designed to examine the association between obesity and ErE. The study population comprised 2,795 consecutive patients who underwent esophagogastroduodenoscopy (EGD) at Nassau University Medical Center (NUMC) in East Meadow, NY, between January 2011 and March 2013. NUMC, a 631-bed multidisciplinary teaching hospital, is part of the North Shore-Long Island Jewish (NSLIJ) Health System.

In order to capture patients with significant symptoms for our study population and to maximize strength of our study, 544 patients were excluded for one or more of the following reasons: age <18 years, incomplete upper endoscopy, procedures performed for gastrostomy or nasogastric tube placement, foreign body removal, endoscopy performed after ingestion of caustic substance, removal of prior placed stents, presence of esophageal varices, known history of gastroesophageal malignancy or procedures performed in asymptomatic patients with significant family history of gastroesophageal cancer, surveillance endoscopy performed for either esophageal varices or Barrett's esophagus, prior gastroesophageal surgery, endoscopy performed prior to bariatric surgery in completely asymptomatic patients, individuals with BMI <18 kg/m^2^, massive hematemesis precluding visualization of the esophagus, and inability to retrieve adequate patient information. The final study population comprised 2251 individuals. In the analysis looking at the effects of BMI on ErE by race groups, only Whites, Blacks, and Hispanics were considered and other race/ethnic groups were excluded from analyses due to sparse numbers. This analysis included 2107 individuals. The study protocol was approved by the NSLIJ Health System Institution Review Board.

### 2.2. Data Collection

Pertinent patient data retrieved from chart review included age, sex, race, height in inches, weight in kilograms, clinical indication for upper endoscopy, endoscopic findings, smoking and alcohol use, use of nonsteroidal anti-inflammatory drug (NSAID) and acid suppression therapy, presence of diabetes mellitus, and histopathology reports. Race was identified and categorized as Caucasians, Blacks, Hispanics, and others. BMI was calculated by dividing weight (kg) by height squared (m^2^) and was categorized as normal (18.5–24.9 kg/m^2^), overweight (25–29.9 kg/m^2^), grade I obesity (30–34.9 kg/m^2^), grade II obesity (35–39.9 kg/m^2^), and grade III obesity (>40 kg/m^2^). For the analysis on race/ethnicity and the effects of BMI on ErE, overweight, Class I, and Class II individuals were combined to form one group due to sparse data in each of the strata. Patients were classified by smoking status as current smokers, former smokers, and nonsmokers (lifetime abstainers). Individuals were classified by alcohol consumption status as current, former, and never drinkers (lifetime abstainers). Severity of ErE was graded A to D according to the Los Angeles (LA) classification of esophagitis [31]. In addition, presence of hiatal hernia diagnosed by upper endoscopy was documented.

### 2.3. Statistical Analysis

Values are presented as number (percent) or mean ± standard deviation. Risk factors, including demographic, metabolic, and lifestyle parameters, were evaluated for their association with ErE using univariate analysis (categorical variables were compared with the *χ* ^2^ test and continuous variables were compared with the *t*-test). Odds ratio (OR) and 95% confidence intervals (95% CIs) were calculated for each variable. The association between BMI and ErE was calculated using multivariate analysis logistic regression. The models were adjusted for age, race/ethnicity, sex, alcohol use, smoking, NSAID use, acid suppression therapy, presence of peptic ulcers,* Helicobacter pylori* (*H. pylori*) positivity on histopathological examination, and presence of hiatal hernia. Effect of modification was assessed by using interaction terms in the regression models. To assess trends in the associations between BMI and either sex or race/ethnicity and ErE, stratified analysis by sex and race was conducted and a test for trend was conducted by modeling BMI as a continuous variable in the regression models. A *p* value of <0.05 (two-sided) was considered statistically significant. All statistical analysis was carried out using the SAS version 9.3 (SAS Institute, Cary, NC, USA).

## 3. Results

### 3.1. Demographics

Among cases (those with ErE), 53.71% were female (*n* = 355), while 63.65% (*n* = 1012) of controls were female (Table 1). The difference between cases and controls in gender breakdown was statistically significant (*p* < 0.01). Male gender was associated with increased odds of ErE. Cases and controls did not differ in mean age. Cases had a higher mean BMI (29.85 kg/m^2^, SD 6.45) than controls (27.77 kg/m^2^ SD 8.57) and the difference was statistically significant (*p* < 0.01). There was a significant difference between cases and controls in racial/ethnic breakdown (*p* < 0.01). For the cases, 28.14% (*n* = 186) were White, 22.9% (*n* = 148) were Black, 42.51% (*n* = 281) were Hispanic, and the rest were other races. For the controls, 17.42% (*n* = 277) were White, 27.99% (*n* = 445) were Black, 48.43% (*n* = 770) were Hispanic, and the rest were other races. Black and Hispanic race/ethnicity were associated with lower odds of ErE (OR = 0.49, 95% CI 0.38–0.64, *p* < 0.01, and OR = 0.54, 95% CI 0.43–0.68, *p* < 0.01), respectively. Cases and controls differed significantly in the breakdown of alcohol use, smoking, hiatal hernia, acid suppression therapy, and* H. pylori* infection (*p* < 0.05 for all variables). Smoking, use of alcohol, and hiatal hernia acid suppression therapy were all associated with increased odds of ErE. Cases and controls did not differ in their NSAID use or presence of either diabetes or peptic ulcer.

### 3.2. Obesity and Erosive Esophagitis

Table 2 shows the odds of having ErE according to BMI. Those in the overweight category had higher odds of ErE than those with normal BMI (OR = 2.66, 95% CI 2.01–3.52, *p* < 0.01), as did those in the Class I obesity group (OR = 3.62, 95% CI 2.64–4.95, *p* < 0.01), the Class II obesity group (OR = 2.84, 95% CI 1.83–4.38, *p* < 0.01), and the Class III obesity group (OR = 6.26, 95% CI 3.82–10.28, *p* < 0.01). Though the odds ratio for the Class II obesity group was lower than the odds ratio for the Class I obesity group, the trend for higher odds ratios with higher classes of obesity was statistically significant (*p* < 0.01). Overweight and Class I and II obese individuals combined also had higher odds of ErE than controls (OR = 2.89, 95% CI 2.28–3.65, *p* < 0.01) as did all the obesity categories combined compared to controls (OR = 3.04, 95% CI 2.41–3.84, *p* < 0.01).

### 3.3. Effect of Obesity on Erosive Esophagitis by Gender

Table 3 shows the effects of obesity and gender on ErE by comparing various BMI gender combinations against normal weight females. The odds of a Class III obese male having ErE as compared to a normal weight female were increased (OR = 8.24, 95% CI 3.72–18.23, *p* < 0.01) as were the odds of ErE in a Class III obese female compared to a normal weight female (OR = 5.50, 95% CI 3.20–9.46). Though the odds ratio for males was higher than the odds ratio for females, the confidence interval for males was wide and the interaction term between gender and BMI was not statistically significant. There was a significant trend in increasing odds of ErE with increasing BMI in both males and females (*p* < 0.01).

### 3.4. Effect of Obesity on Erosive Esophagitis by Race

Table 4 shows the effects of obesity and race/ethnicity on ErE by comparing various BMI race/ethnicity combinations against normal weight Whites. Normal weight Blacks (OR = 0.46, 95% CI 0.27–0.79, *p* < 0.01) have lower odds of ErE than normal weight Whites, while the odds ratio for Class III Hispanics compared to normal weight Whites was not statistically significant. The interaction term between race/ethnicity and BMI was not statistically significant. There was a significant trend of increasing odds ratios with increasing BMI in all ethnicities (*p* < 0.01).

## 4. Discussion

To our knowledge, this is the first epidemiologic study examining the association between obesity and ErE in a racially diverse community with a significant Hispanic and Black population. Additionally, we assessed the association between obesity and ErE in each gender. There was no statistically significant interaction between race/ethnicity and BMI or gender and BMI.

There was a significant trend of increasing odds of ErE with BMI in every race/ethnicity and in each gender. The exact pathophysiological mechanism between obesity and GERD is not known [10]. It has been hypothesized that increased abdominal adipose tissue associated with obesity may cause extrinsic gastric compression which in turn leads to augmentation of intragastric pressures along with relaxation of the lower esophageal sphincter (LES) and possible anatomic disruption of the gastroesophageal junction resulting in a hiatal hernia [5, 31]. It has also been suggested that obesity is associated with more frequent transient LES relaxations (TLESRs), increased gastric acid production, and esophageal acid exposure [8, 32, 33]. Additionally, increased visceral fat has been strongly linked to greater secretion of proinflammatory cytokines such as interleukin-6 as well as lower levels of adiponectin which has an antiproliferative effect [5, 34].

In addition to indicating a positive association between obesity and erosive reflux esophagitis, our study illustrates that increasing levels of BMI are associated with a proportional increase in the OR of ErE. Interestingly, the OR of ErE among individuals with a BMI of 35–39.9 kg/m^2^ was lower than those with a BMI of 30–34.9 kg/m^2^. These results should be interpreted with caution and could either reflect a patient selection bias or a consequence of unaccounted confounding factors. Additionally the effect of undocumented weight changes should be considered as a likely explanation for these findings. It is plausible that a high BMI caused ErE, leading to weight reduction and thus weakening the BMI-ErE association during weight measurement at the interview. It is also possible that recent weight changes prior to the individual's anthropometric measurements moved the subject from one class of obesity to another, thus skewing our results. Finally, endoscopist variation in interpreting subtle esophageal findings could also have contributed to this variation. In our study, the OR for acid reflux related esophageal injury was 1.5 times higher in men, which is consistent with other published data and a recently published meta-analysis [23, 24, 28, 34, 35]. This gender variation may be explained by the distribution of body fat which tends to be more visceral than truncal in men as compared to women [5].

Although it has been suggested that acid reflux disease and its related adverse effects are more common among Caucasians in the United States, data has been inconclusive since these studies did not include a significant Hispanic or Black population. In addition, the majority of existing data examining the association between obesity and ErE originated in Asian countries where the prevalence of GERD and ErE is lower than in Western countries [24]. Explanations offered for these ethnic differences are differences in parietal cell mass and gastric acid secretion, genetic disparities including ethnic variation in body composition and distribution of visceral adipose tissue, differences in dietary habits, and ethnic variations in symptom reporting [5, 9, 10, 29, 30, 36]. In our study, after adjusting for multiple confounding factors, it was demonstrated that, compared to Caucasians, Hispanics and Blacks were at a lower risk of ErE. However when individuals were stratified by BMI and race/ethnicity we did not demonstrate a significant interaction possibly due to smaller sample sizes.

Our study has several strengths including an ethnically diverse patient population. In addition to physical examination being performed by trained personnel, we used endoscopic evaluation and valid anthropometric measurements instead of a questionnaire and self-reported measurements. Also we categorized our subjects into different classes of obesity, defined strict exclusion criteria, and adjusted for several confounders, which significantly increased the quality our data. This study also has many limitations that need to be considered when interpreting the findings. Firstly, retrospective case control single center studies have the inherent weakness of not being able to infer causality. In addition, it is subject to certain bias such as selection bias which can arise from various factors including ethnic variations in referral for endoscopy. Population based randomized control trials are essential to eliminate these risks. The effect of residual confounding such as diet, physical activity, changes in weight, socioeconomic status, and geographic distribution should be considered. Further, we did not quantify smoking and alcohol consumption. Drugs and hormones that may potentially affect the interaction between obesity and ErE such as estrogen were not included as confounding factors in calculations due to the retrospective nature of our study and nonuniform data collection regarding oral contraceptive or hormone replacement therapy. Third, our study population did not contain adequate numbers of individuals from other racial backgrounds. Fourth, there is controversy regarding the optimal method to measure obesity. Some data shows that abdominal visceral adipose tissue measurement may be better than BMI or waist circumference as a predictor of GERD [23, 35, 37].

In conclusion, our study illustrates the strong positive association between BMI and ErE in a multiethnic community and in both genders. Physicians should be cognizant of the relationship between obesity and ErE in multiethnic patient populations; future studies could achieve larger sample sizes and include patients of other ethnicities through pooled analysis with other study sites.

## Figures and Tables

**Table 1:** Comparisons of patient characteristics based on endoscopic presence of erosive esophagitis.

	Normal esophagus (*n* = 1590)	Erosive esophagitis (*n* = 661)	OR (95% CI)^*∗*^	*p* value^*∗*^
Gender				
Females	1012 (63.65)	355 (53.71)	1	
Males	578 (36.35)	306 (46.29)	1.51 (1.26–1.81)	<0.01
Age (mean ± SD)	52.64 ± 15.49^*∗∗*^	52.97 ± 14.37^*∗∗*^		0.61
Mean BMI kg/m^2^ (SD)	27.99 (8.57)	29.85 (6.45)		<0.01
Race				
Whites	277 (17.42)	186 (28.14)	1	Chi-square <0.01
Blacks	445 (27.99)	148 (22.39)	0.49 (0.38–0.64)	<0.01
Hispanics	770 (48.43)	281 (42.51)	0.54 (0.43–0.68)	<0.01
Others	98 (6.16)	46 (6.96)		
Alcohol use				
Never use	1186 (76.96)	421 (66.83)	1	
Former use	86 (5.58)	49 (7.78)	1.61 (1.11–2.32)	0.01
Current use	269 (17.46)	160 (25.4)	1.68 (1.34–2.10)	<0.01
Smoking				
Never smoker	1196 (76.23)	440 (68.01)	1	
Former smoker	140 (8.92)	70 (10.82)	1.36 (1.00)	0.05
Current smoker	233 (14.85)	137 (21.17)	1.60 (1.26–2.03)	0.01
Hiatal hernia	374 (23.54)	189 (28.59)	1.30 (1.06–1.60)	0.01
NSAID use	377 (23.73)	167 (25.26)	1.09 (0.88–1.34)	0.44
Acid suppression therapy	716 (45.09)	244 (36.91)	0.71 (0.59–0.86)	<0.01
Diabetes mellitus	273 (17.18)	110 (16.64)	0.96 (0.76–1.23)	0.76
*H. pylori* infection	436 (29.68)	126 (21.36)	0.64 (0.51–0.81)	<0.01
Peptic ulcer	274 (17.23)	128 (19.36)	1.15 (0.91–1.46)	0.23

^*∗*^Adjusted for age, race, sex, alcohol use, smoking, NSAID use, acid suppression therapy, presence of peptic ulcers, *Helicobacter pylori* (*H. pylori*) positivity on histopathological examination, and presence of hiatal hernia.

^*∗∗*^Age documented at the time of the initial endoscopic intervention.

**Table 2:** Association between obesity and erosive esophagitis.

Body mass index	Normal esophagus (*n* = 1590)	Erosive esophagitis (*n* = 661)	OR (95% CI)^*∗*^	*p* value	*p* for trend
Normal weight (18.5–24.9 kg/m^2^)	569 (35.79)	121 (18.31)	1		
Overweight (25–29.9 kg/m^2^)	563 (35.41)	272 (41.15)	2.66 (2.01–3.52)	<0.01	<0.01
Class I obesity (30–34.9 kg/m^2^)	298 (18.74)	168 (25.42)	3.62 (2.64–4.95)	<0.01	
Class II obesity (35–39.9 kg/m^2^)	106 (6.67)	46 (6.96)	2.84 (1.83–4.38)	<0.01	
Class III obesity (>40 kg/m^2^)	54 (3.4)	54 (8.17)	6.26 (3.82–10.28)	<0.01	
Overweight or obese Classes I-II combined	967 (60.82)	486 (73.52)	2.89 (2.28, 3.65)	<0.01	
Overweight or obese Classes I–III combined	1021 (64.32)	540 (81.69)	3.04 (2.41, 3.84)	<0.01	

^*∗*^Adjusted for age, race, sex, alcohol use, smoking, NSAID use, acid suppression therapy, presence of peptic ulcers, *Helicobacter pylori* (*H. pylori*) positivity on histopathological examination, and presence of hiatal hernia.

**Table 3:** Joint effects of obesity and gender on erosive esophagitis^*∗*^.

	Normal esophagus (*n* = 1590)	Erosive esophagitis (*n* = 661)	OR (95% CI)^*∗∗*^	*p* value	*p* for trend
Normal weight female^*∗*^	332 (20.88)	64 (9.68)	1		In females *p* < 0.01
Overweight female	346 (21.76)	130 (19.67)	*2.30 (1.63–3.26)*	*<0.01*	
Class I obesity female	220 (13.84)	96 (14.52)	*2.75 (1.90–3.98)*	*<0.01*	
Class II obesity female	73 (4.59)	29 (4.39)	*2.51 (1.49–4.22)*	*<0.01*	
Class III obesity female	41 (2.58)	36 (5.45)	*5.50 (3.20–9.46)*	*<0.01*	

Normal weight male	237 (14.91)	57 (8.62)	1.06 (0.71–1.60)	*0.76*	In males *p* < 0.01
Overweight male	217 (13.65)	142 (21.48)	3.49 (2.45–4.96)	*<0.01*	
Class I obesity male	78 (4.91)	72 (10.89)	4.90 (3.18–7.53)	*<0.01*	
Class II obesity male	33 (2.08)	17 (2.57)	2.68 (1.38–5.17)	*<0.01*	
Class III obesity male	13 (0.82)	18 (2.72)	8.24 (3.72–18.23)	*<0.01*	

Overweight or obese Classes I-II female	639 (40.19)	255 (38.58)	2.48 (1.81–3.39)	*<0.01*	In females *p* < 0.01
Overweight or obese Class III female	41 (2.58)	36 (5.45)	*5.50 (3.20–9.46)*	*<0.01*	

Overweight or obese Classes I-II male	328 (20.63)	231 (34.95)	3.74 (2.70–5.19)	*<0.01*	In males *p* < 0.01
Overweight or obese Class III male	13 (0.82)	18 (2.72)	8.24 (3.72–18.23)	*<0.01*	

Overweight or obese Classes I–III female	680 (42.77)	291 (44.02)	2.66 (1.95–3.62)	*<0.01*	
Overweight or obese Classes I–III male	341 (21.45)	249 (37.67)	3.87 (2.80–5.36)	*<0.01*	

^*∗*^Reference category for all regression models in table.

^*∗∗*^Adjusted for age, race, sex, alcohol use, smoking, NSAID use, acid suppression therapy, presence of peptic ulcers, *Helicobacter pylori* (*H. pylori*) positivity on histopathological examination, and presence of hiatal hernia.

**Table 4:** Joint effects of obesity and ethnicity/race^*∗*^ on erosive esophagitis.

	Normal esophagus (*n* = 1492)	Erosive esophagitis (*n* = 615)	OR (95% CI)^*∗∗*^	*p* value	*p* for trend
Normal weight Whites^*∗*^	129 (8.65)	46 (7.48)	1		*<0.01*
Overweight or Classes I-II obese Whites	136 (9.12)	125 (20.33)	*3.01 (1.96–4.62)*	*<0.01*	
Class III obesity Whites	12 (0.80)	15 (2.44)	*4.28 (1.82–10.08)*	*<0.01*	
Normal weight Blacks	178 (11.93)	27 (4.39)	0.46 (0.27–0.79)	*<0.01*	*<0.01*
Overweight or Classes I-II obese Blacks	243 (16.29)	101 (16.42)	1.53 (1.00–2.35)	*0.05*	
Class III obesity Blacks	24 (1.61)	20 (3.25)	3.30 (1.63–6.69)	*<0.01*	
Normal weight Hispanics	222 (14.88)	37 (6.02)	*0.66 (0.39–1.10)*	*0.11*	*<0.01*
Overweight or Classes I-II Hispanics	532 (35.66)	227(36.91)	*1.83 (1.21–2.75)*	*<0.01*	
Class III obesity Hispanics	16 (1.07)	17 (2.76)	*5.08 (2.30–11.23)*	*<0.01*	

^*∗*^Blacks, Whites, and Hispanics included in analysis.

^*∗∗*^Adjusted for age, race, sex, alcohol use, smoking, NSAID use, acid suppression therapy, presence of peptic ulcers, *Helicobacter pylori* (*H. pylori*) positivity on histopathological examination, and presence of hiatal hernia.
